# Blue Zones, an Analysis of Existing Evidence through a Scoping Review

**DOI:** 10.14336/AD.2025.0461

**Published:** 2026-05-18

**Authors:** Cristina Candal-Pedreira, Julia Rey-Brandariz, Lucía Martín-Gisbert, Ana Teijeiro, Guadalupe García, Alberto Ruano-Ravina, Mónica Pérez-Ríos

**Affiliations:** ^1^Department of Preventive Medicine and Public Health, University of Santiago de Compostela, Santiago de Compostela, Spain.; ^2^Health Research Institute of Santiago de Compostela (*Instituto de Investigación Sanitaria de Santiago de Compostela-IDIS*), Santiago de Compostela, Spain.

**Keywords:** Epigenesis, Genetic, DNA Methylation Aging, Biological Clocks, Biomarkers

## Abstract

“Blue Zones” refer to geographical regions with a high proportion of centenarians. This review aims to identify and synthesize scientific evidence regarding Blue Zones and their characteristics. A scoping review was conducted following PRISMA-ScR guidelines. Systematic bibliographic searches were performed across major scientific databases up to February 2025. Studies were included if they investigated regions which the authors identified as Blue Zones. Non-naturally occurring Blue Zones were excluded. A narrative synthesis of the evidence was carried out. Sixty-five records were included, identifying ten regions: Ogliastra (Sardinia, Italy), Ikaria (Greece), Cilento (Italy), one municipality in The Netherlands, Menorca (Spain), Okinawa (Japan), Rugao (China), Nicoya (Costa Rica), Martinique/Guadeloupe (French overseas regions in the Caribbean) and Loma Linda (California, United States). Longevity indicators revealed that Ogliastra, Okinawa and Nicoya exhibit higher longevity compared to national averages. Ikaria and Cilento show high life expectancy, the municipality in The Netherlands show a higher proportion of exceptional longevous population than other municipalities, whereas Guadeloupe/Martinique present a higher prevalence of supercentenarian deaths than metropolitan France. Data from Menorca remain inconclusive, and Rugao has fewer centenarians. No studies have been conducted in Loma Linda. Factors associated with longevity include diet, physical activity, climate, genetic factors and geographical isolation. The literature indicates that certain geographical areas demonstrated a higher percentage of centenarians than adjacent areas. While regions such as Okinawa, Ogliastra and Nicoya are well-characterized as Blue Zones, others remain under investigation, and some currently lack sufficient scientific evidence.

## INTRODUCTION

Blue Zones have been defined as geographical regions where extreme longevity has been observed [1], characterized by a higher prevalence of centenarians compared to adjacent areas. Currently, scientific literature appears to be consistent in identifying four Blue Zones: Ogliastra (Sardinia, Italy), Okinawa (Japan), Nicoya (Costa Rica) and Ikaria (Greece) [1].

The term 'Blue Zone' was originally coined by Michel Poulain, who, in 2004, used a blue marker to demarcate the first Blue Zone identified by his team on the central-east coast of the island of Sardinia, Italy [2]. In 2005, the trademark “Blue Zones®” was registered by Dan Buettner, following the successful dissemination of the concept through his travel articles, which were published in the National Geographic [3]. This trademark has since evolved into a profitable brand that promotes lifestyles based on 9 principles to follow (the “Power 9®” criteria), which are copyrighted under the name “Power 9®” by the company Blue Zone LLT [4].

The demographic definition of a Blue Zone should adhere to a homogeneous and transparent methodology, to ensure scientific rigor. The methodology was initially proposed by Michel Poulain and was applied to the first Blue Zone, located in Ogliastra, Sardinia. The methodology for identifying Blue Zones is based on the following four steps [1, 5]: 1) analysis of birth certificates of individuals over 100 years old, 2) verification of the continued existence of these individuals and the absence of registration errors, 3) comparison of the percentage of centenarians in the potential Blue Zone with that of adjacent areas, and 4) geographic definition of the Blue Zone.

The proposed methodology relies on the calculation of several longevity indicators, such as the percentage of centenarians or the Extreme Longevity Index (ELI) [5, 6]. The ELI is calculated as the percentage of people born in a 20-year period who reach 100 years of age. This index is expressed per 10,000 inhabitants and is defined as the probability that any person born during the defined period in the area of interest reaches 100 years of age. The application of a Gaussian smoothing technique is then employed to delineate the area where the ELI attains its maximum value. In some cases, two areas were identified and are known as the “restricted Blue Zone” and the “extended Blue Zone”- where the excess of centenarians exists but is less pronounced [2].

The scientific literature on the Blue Zones has increased considerably in recent years. Therefore, the aim of this study is to identify which regions have been defined as Blue Zones by the scientific literature and to ascertain their characteristics.

## METHODS

A scoping review was conducted in accordance with the recommendations of the PRISMA-ScR (Preferred Reporting Items for Systematic Reviews and Meta-Analyses - extension for scoping reviews) [7].

### Search strategy

A comprehensive literature search was conducted in April 2024 and updated until February 2025 in the following databases: MEDLINE (PubMed), EMBASE, PsycINFO, Web of Science (WoS) and SCOPUS. The search strategy was designed by three expert researchers in the field and was applied consistently across all databases.

To identify relevant records, a search strategy was employed that combined Medical Subject Headings (MeSH) terms and free terms related to Blue Zones and extreme longevity. The search strategy is detailed in Supplementary Table 1. A manual review of the references of the selected studies was performed to identify additional papers. No restrictions were applied in terms of the country where the study was conducted, study period, study design or language.

EndNote was employed to manage records and remove duplicates, and study selection was performed using Rayyan.

### Inclusion and exclusion criteria

This scoping review included studies published in a scientific journal with peer review that assessed the identification and/or characterization of extreme longevity zones, as referred to by the authors as Blue Zones. Regions which attempt to imitate factors related to longevity which were identified in naturally occurring Blue Zones (i.e. Malvaalan (The Netherlands) or the Beach Cities (California, U.S.)) were excluded from this review.

Studies published in languages other than English, Spanish, Portuguese, Italian, and French were excluded. This language selection aimed to prioritize the inclusion of region-specific data while mitigating anglocentric biases. This review excluded conference abstracts, letters to the editor and opinion articles as well as case series and simulation studies. Retracted publications were also excluded.

### Selection of studies

Following the elimination of duplicate records, all authors independently peer-reviewed the titles and abstracts of all articles identified through the literature search. Those studies considered potentially relevant were subsequently subjected to a full-text review, also conducted independently in pairs. Discrepancies in study selection or data interpretation were resolved by consensus.

### Analysis of the evidence

The Blue Zones identified were classified as 'well characterized', 'under investigation' and 'excluded' based on the available evidence. ‘Well-characterized' Blue Zones are those with longevity indicators calculated using official data that confirm the extreme longevity, with several published studies on the factors associated with longevity, and with sustained observation of longevity patterns over the years. Blue Zones 'under investigation' are those with anecdotal reports, initial demographic data or observational studies suggesting exceptional longevity, but without rigorous validation published in a scientific journal. Blue Zones 'excluded' are those with insufficient demographic data or studies that refute extreme longevity. The results are presented as a narrative synthesis of the evidence. For each region, detailed information was collected on its location, its characteristics and the process of its identification. In addition, factors associated with the extreme longevity of its inhabitants were synthesized. If any extreme longevity indicators were calculated in the study, they were also collected.

## RESULTS

### Search results

**Figure 1.**
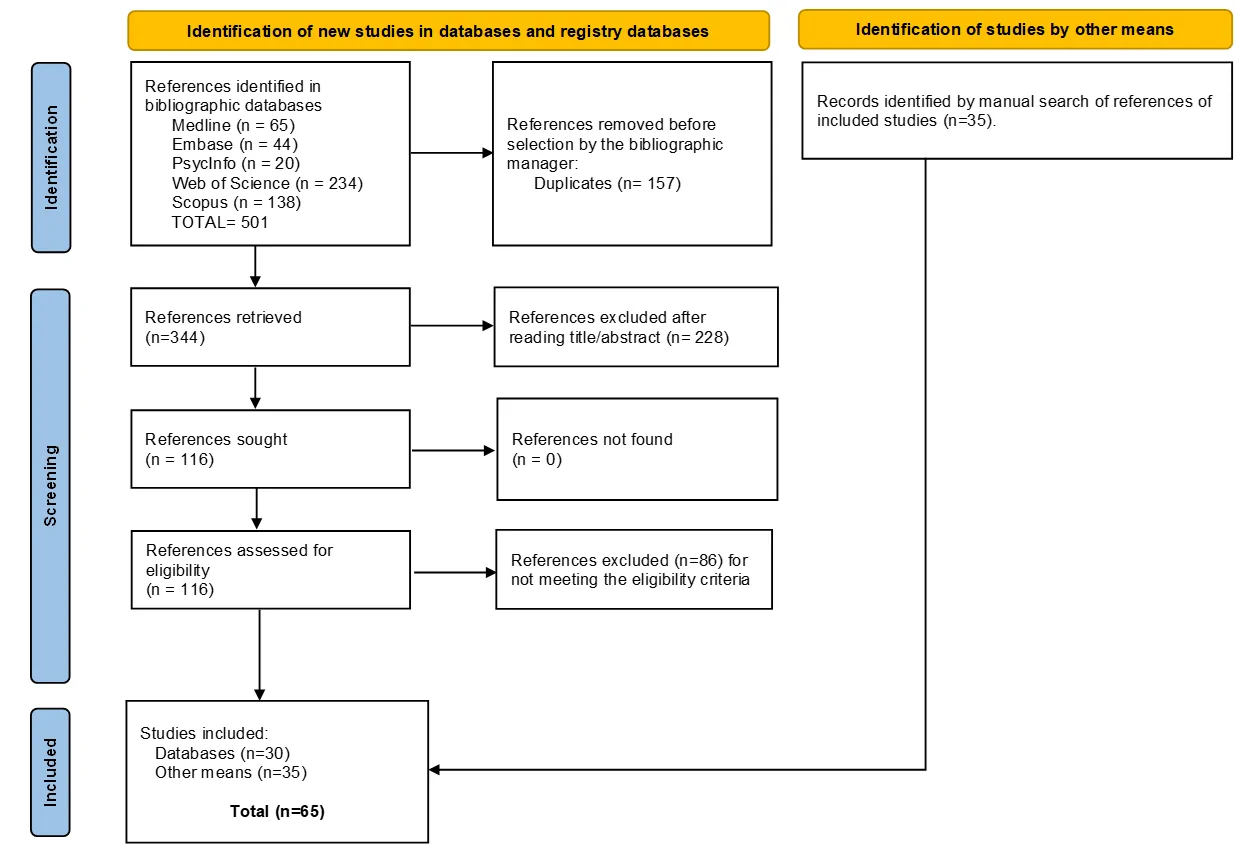
Flow-chart of study selection.

The literature search yielded a total of 344 potentially relevant studies. Following a review of the titles and abstracts, 116 were selected for full-text reading. Of these, 86 were excluded due to non-compliance with the established eligibility criteria, while 30 were ultimately included. Additionally, a manual review of the references of the selected studies led to the identification and inclusion of 35 additional studies. The final number of studies included in the scoping review was 65. The selection process is detailed in Figure 1.

### Results of the included articles

Ten geographical regions were referred to as Blue Zones or potential Blue Zones by the authors of the articles included.

In Europe, the following regions were identified: Ogliastra (Sardinia, Italy) [2, 5, 8-23], Ikaria (Greece) [5, 24-29], Cilento (Italy) [30-32], one municipality in The Netherlands (not specified the name) [33] and Menorca (Spain) [34]; in Asia, Okinawa (Japan) [35-51] and Rugao (China) [52-59]; and in America, Nicoya (Costa Rica) [18, 60-66], Martinique/Guadeloupe (French overseas departments in the Caribbean) [67] and Loma Linda (California, United States) [4]. Table 1 describes the characteristics of each of the regions identified. All these regions lie within a similar latitudinal range and have similar topography. Except for Loma Linda and Rugao, the regions have a medium-low population density, ranging from 35 to 319 inhabitants per square kilometer. Women in all regions exceed 80 years of age in terms of life expectancy. The regions identified have a diversity of socio-economic levels, although five of them are low-income regions.

**Table 1:** Characteristics of the regions identified as Blue Zones.

Region	Location	Latitude	Topography	Surface area	Population	Population density	Life expectancy	Economic situation
**Ogliastra (Sardinia, Italy)**	Island in the Mediterranean Sea, west of Italy	39°52′00″N 9°33′00″E	Mountains and hills	Not available	Not available	69 inhabitants/km^2^	82.5 years (85.2 women, 80 men)	Lower income area, < $20,000 per year, like Sicily and Catania
**Okinawa (Japan)**	Main island of the Ryukyu Islands, south of Japan	26°20′03″N 127°48′21″E	Mountains and hills	1,201 km^2^	145,754 inhabitants	121 inhabitants/km^2^	83.3 years (86.8 women, 79.9 men)	Poorest prefecture in Japan
**Nicoya (Costa Rica)**	Peninsula in northwestern Costa Rica	10°26′00″N 85°24′00″W	Coast, tropical vegetation, mountainous terrain	5,130 km^2^	207,000 inhabitants	Varies by canton	81.9 years (women), 77.3 years (men)	Medium-low position in Costa Rica's human development index
**Ikaria (Greece)**	Island in the eastern Aegean Sea	37°35′00″N 26°10′00″E	Mountains and hills	255.3 km^2^	8,843 inhabitants	35 inhabitants/km^2^	82.3 years (84.9 women, 79.9 men)	Intermediate-low wealth region, < $20,000 per capita annually
**Cilento (Italy)**	Southwestern area of the Italian peninsula	40°18′00″N 15°18′00″E	Coast, hills, mountains	3,500 km^2^	288,000 inhabitants	Not available	Not available	Most economically productive in southern Italy, seventh in the country
**The Netherlands (one municipality defined as N1)**	The municipality is not defined	Not available	Not available	Not available	Not available	Not available	Not available	Not available
**Martinique/Guadeloupe (France)**	Islands in the Caribbean, north of St. Lucia and south of Dominica	14°38'29.5″ N 61°1.45' O	Volcanic origin, mountains	1,128 km^2^ (Martinique), 1,628 km^2^ (Guadeloupe)	343,195 inhabitants (Martinique), 375,106 inhabitants (Guadeloupe)	324 inhabitants/km^2^ (Martinique), 221 inhabitants/km^2^ (Guadeloupe)	83.1 global, 85.7 women and 79.5 men (Martinique), 82.2 global, 85.7 women and 78.4 men (Guadeloupe)	Per capita income is lower than that of metropolitan France and other overseas departments
**Menorca (Spain)**	Archipelago of the Balearic Islands, easternmost island	39°58′00″N 4°05′00″E	Plains and hills with little vegetation	702 km^2^	99,000 inhabitants	142 inhabitants/km^2^	84.4 years (86.6 women, 82.3 men)	Highest economic growth in the Balearic Islands, GDP per capita > €30,000
**Rugao (China)**	Northeastern China, Jiangsu province	32°22′25″N 120°34′00″E	Flat terrain with low hills	1,531 km^2^	1.5 million inhabitants	980 inhabitants/km^2^	Not available	Intermediate position among cities in the province (GDP)
**Loma Linda (California, U.S.)**	San Bernardino County, Southern California	34°02′54″N 117°15′02″W	Valley surrounded by hills, nature reserve	19 km^2^	24,883 inhabitants	1,273 inhabitants/km^2^	78.6 years (81.1 women, 76.1 men)	Median income of $70,685, similar to the U.S. average

GDP: gross domestic product. U.S.: United States

Of the identified regions, Ogliastra, Nicoya and Okinawa were classified as ‘well-characterized’ Blue Zones; Ikaria, Cilento, one municipality in The Netherlands, Martinique/Guadeloupe and Rugao were classified as ‘under investigation’ Blue Zones; and Menorca and Loma Linda were classified as ‘excluded’ Blue Zones.

Table 2 shows a summary of longevity indicators and factors related to longevity for each of the identified regions. The supplementary material contains detailed information for each region. With respect to indicators, Ogliastra, Okinawa, and Nicoya exhibit high longevity indicators compared to the national average and adjacent regions. However, recent trends in Okinawa and Nicoya show that indicators are starting to resemble those of adjacent areas, attributable to westernization. Ikaria and Cilento also demonstrate high life expectancy. Guadeloupe and Martinique, show a high supercentenarian death prevalence (21 and 24 per million inhabitants, respectively), exceeding rates in metropolitan France (3 per million). A recent study in The Netherlands identified one municipality, referred to as N1, as a relative Blue Zone, that showed high longevity indicators, with a high proportion of participants reaching exceptional ages (95 years and over) and a longer life expectancy. The data from Menorca are inconclusive due to its small population size, and Rugao exhibits stable longevity until 99 years old but a lower number of centenarians. No specific longevity studies have been conducted in Loma Linda.

**Table 2:** Identification, longevity indicators and factors related to longevity of regions identified as Blue Zones in the scientific literature.

Region	Identification	Indicators	Factors related to longevity
**Ogliastra (Sardinia, Italy)**	Identified in 1999 by the AKEA study.	High prevalence of centenarians (16.6 vs. 10.0 per 100,000 in Europe). Female/male ratio of 2:1, with the historical province of Nuoro standing out. High longevity indicators in Barbagia of Ollolai. The 1902-1911 cohort showed higher survival rates. No clear trends in longevity across areas in 2012 compared to previous data.	Several factors, including diet, physical activity, and mental health. The traditional diet, which was frugal until 1950, consisted mainly of grains, legumes, and sheep/goat dairy, later shifting to higher carbohydrate, olive oil, and meat consumption. Physical activity is associated with longevity in the region, and mild overweight is common among the population. Cultural traditions and good mental health also contribute to the overall well-being of the people.
**Okinawa (Japan)**	Identified in 1976 after the 1975 census. Studies such as the Okinawa Centenarian Study.	Number of centenarians increased from 37 (1975) to 1,271 (2022). High prevalence of centenarians (35.5 vs. 5.1 per million in Japan). Female predominance in longevity. High life expectancy but trending closer to Japan due to post-WWII westernization. Recent increase in mortality rates in Okinawa.	Traditional diet, genetics, and physical activity. The Okinawan diet is low in calories but nutrient-rich, high in antioxidants, lean pork, and vegetables. The FOXO3 gene has been associated with longevity in the population. Physical activity is a key component of daily life, particularly through farming, and social and cultural factors, along with a warm climate, further contribute to longevity.
**Nicoya (Costa Rica)**	Identified in 2005 during the CRELES project.	High proportion of centenarians (1:1.38 male/female ratio in 2017). Lower male mortality rates than the national average for those over 60. High longevity in cohorts born before 1930, with a decline in recent cohorts. The extreme longevity zone has shrunk geographically.	Combination of diet, physical activity, and environmental factors. The local diet is rich in calories, carbohydrates, protein, and fiber, while being low in processed foods and red meat. High physical activity levels are rooted in traditional farming practices and daily movement, and the population enjoys a youthful immunological profile. The region also benefits from good air quality and high light exposure.
**Ikaria (Greece)**	Identified in 2009 through the IKARIA study.	High life expectancy for those over 65 compared to the rest of Greece. The age of the oldest inhabitants (>90 years old) was validated. Few studies are available on mortality. Specific indicators such as ELI have not been analyzed.	Family solidarity, diet, physical activity, and sleep quality. The Mediterranean diet, rich in olive oil, fruits, and vegetables, with low consumption of sweets and red meat, is central to the Ikarian lifestyle. Regular physical activity, often in the form of farming and walking, and frequent naps contribute to good sleep quality. Strong social interactions and family ties are vital to mental and emotional health, while the population experiences a lower prevalence of cardiovascular risk factors, multimorbidity, polypharmacy, and depression.
**Cilento (Italy)**	Several recent studies (2022-2024) analyze longevity indicators and environmental factors.	Longevity indicators studied: centenarian rate, ratio of people over 85 and 90 years old, longevity and centenarian indexes.	Environmental factors including altitude, climate, and water quality (low in heavy metals). Indicators show correlation between environmental factors and longevity.
**The Netherlands (one municipality defined as N1)**	Studied in 2019 through the Longitudinal Aging Study Amsterdam.	N1 (municipality) has a cumulative proportion of exceptional longevous participants of 8.3%. Comparison with other municipalities.	Favorable genetics, a healthy lifestyle, and a walkable environment. The population benefits from a favorable genetic makeup and healthier lifestyle habits, such as less smoking, lower alcohol consumption, and a higher fruit intake. Regular biking and a strong sense of religiousness and community participation in activities like singing and paid work further contribute to the region’s longevity. The more walkable and livable environment also supports an active and healthy lifestyle.
**Martinique/Guadeloupe (France)**	A study validated the age of super-centenarians who died between 1988 and 2016.	The prevalence of supercentenarian deaths in these regions was found to be 7 to 8 times higher than in metropolitan France.	Genetic selection from extreme conditions during slavery, high maternal fertility, and longer-lived siblings, suggesting a hereditary component.
**Menorca (Spain)**	A single study analyzes whether Es Migjorn Gran and surrounding areas could be a Blue Zone.	The study concludes there is not enough evidence to classify Menorca as a Blue Zone. Centenarian records are inconclusive due to the small population size.	No scientific evidence identified.
**Rugao (China)**	Studied since 2007-2008 by the Rugao Longevity and Ageing Study (RuLAS) as a potential Blue Zone.	A stable longevity zone exists in the central region, where people are more likely to reach 90-99 years, but fewer reach 100. Progressive decrease in male-to-female ratio with increasing age.	No scientific evidence identified.
**Loma Linda (California, U.S.)**	Identified by Dan Buettner in 2005.	No published studies on longevity or specific indicators in Loma Linda.	Population influenced by Seventh-day Adventists. Vegetarian diet, no alcohol consumption, moderate physical activity, spirituality.

WWII: World War II. ELI: Extreme Longevity Index. U.S.: United States.

Several factors associated with longevity have been identified in the regions examined in this review, and some factors are replicated across these regions. One of these factors is related to diet. Okinawa and Nicoya exhibit a pattern that is consistent with traditional diets, which include the consumption of vegetables, pork and antioxidants. These diets have been linked to a low incidence of cardiovascular disease and cancer in these regions. Ogliastra and Ikaria stand out for their adherence to frugal, Mediterranean diets, characterized by high consumption of olive oil, fruits and vegetables and low consumption of red meat and processed foods. In the specific case of Ogliastra, the traditional diet is closely associated with transhumance and the pastoral economy. A recent study conducted in Sardinia has identified that mild overweight in nonagenarians is associated with increased life expectancy. In N1 (The Netherlands), there is less alcohol and tobacco consumption, more fruit consumption and more use of bicycles for transport than in near municipalities. The relationship between diet and soil characteristics has been identified in several regions. For instance, Okinawa, Ikaria, and Ogliastra are volcanic and karstic islands and, in some of them, vegetation is sparse, resulting in lower soil fertility compared to other areas.

Geographical isolation also seems to be a relevant factor. Sardinia, for example, shows a high number of centenarians in isolated areas such as the historical province of Nuoro, which could be linked to inbreeding and protection against disease. However, there is mixed evidence regarding this factor, as one study found no differences in genetic polymorphisms related to longevity between nonagenarians living in the Blue Zone and controls living in a nearby area. In Guadeloupe and Martinique, the evidence suggests that genetic selection due to extreme conditions during slavery may have favored descendants with robust longevity genes. The absence of similar longevity patterns in regions such as La Réunion, where slavery persisted until 1848, challenges this theory. The observed longevity in Guadeloupe and Martinique is linked to family patterns. These include high maternal fertility, lower fertility among the supercentenarians themselves, and exceptional sibling longevity. N1 residents may have a greater genetic predisposition to longevity, as evidenced by a favorable polygenic risk score and the presence of the apolipoprotein E-epsilon2 allele. Additionally, this municipality has a high population stability, a factor that may contribute to the preservation of genetic factors associated with longevity.

In addition, air quality and climate may be relevant environmental factors linked to longevity in regions such as Okinawa, Nicoya and Cilento, and the fact that all of them share a similar latitude.

There are other regions that do not present scientific evidence on factors associated with longevity. Menorca and Rugao have no analysis of factors related to longevity. Loma Linda, influenced by the Seventh Day Adventist community, has no specific studies on longevity.

## DISCUSSION

The existing literature seems to indicate that there are certain geographical areas in different parts of the world where there is a higher percentage of centenarians compared to adjacent areas. On the one hand, there are areas that have consolidated status as Blue Zones from a demographic and epidemiological point of view. These regions include Okinawa (Japan), Ogliastra (Sardinia, Italy) and Nicoya (Costa Rica). On the other hand, there are regions under investigation, such as Ikaria (Greece), Cilento (Italy), Martinique/Guadeloupe (France), one municipality in The Netherlands and Rugao (China), regions that have been discarded as Blue Zones for now, such as Menorca (Spain), and regions which lack scientific basis, such as Loma Linda (California, United States). The results of this review also show that some Blue Zones may have lost the advantage in terms of longevity in recent decades, as evidenced by the cases of Okinawa and Nicoya.

### Methodology for the identification of a Blue Zone

Characterizing a Blue Zone is complex due to the need for accurate population records, age verification, and migration tracking. Administrative boundaries can be unclear, complicating demographic analysis. A region qualifies as a Blue Zone based on a significant centenarian population, which generally improves longevity indicators. However, in small areas like Menorca [34], limited population size can lead to data instability, possibly explaining why some less populated extreme longevity regions remain uncharacterized.

Loma Linda, California, is often cited as a Blue Zone, but there are no epidemiological studies to confirm its status as a Blue Zone. The only reference to Loma Linda comes from Dan Buettner's review [68], which lacks demographic studies and only includes personal narratives. Buettner later clarified that Loma Linda's inclusion in National Geographic magazine was at the request of his editor, who wanted a Blue Zone in the U.S. [69].

Although Ikaria is widely recognized as a Blue Zone, its classification is largely based on studies assessing lifestyle rather than demographic data. Available information on extreme longevity on the island is limited, in part due to its small population size, which may result in some years without any recorded centenarians. We identified one study by Poulain et al. [5] comparing the Sardinian and Ikarian Blue Zones, using survival probabilities and life expectancy at birth for individuals aged 60–69 in 1970 and 90–99 in 2000. Most of these indicators do not differ as markedly from the rest of Greece as Ogliastra does from the rest of Sardinia. No other demographic indicators were found in the literature apart from the number of nonagenarians. For this reason, we consider it more appropriate to classify Ikaria as a Blue Zone ‘under investigation,’ until further demographic research enables a more robust evaluation of its longevity indicators.” Martinique and Guadeloupe, overseas departments of France, have been lately studied as Blue Zones and a validation of the ages of supercentenarians has been conducted [67]. Poulain's website includes a manuscript that validates the age of centenarians in Martinique, however it has not been published in a scientific journal and therefore could not be included in the present review. This study estimates a higher centenarian prevalence in Martinique than in metropolitan France. The probability of reaching 100 years of age has been increasing in the region, and it has always been higher than in the rest of France. The mortality rate in Martinique for all ages between 90 and 105 is lower than in metropolitan France. The study is available at: https://longevitybluezone.com/the-5-blue-zones/ martinique.

Controversy exists in the scientific community regarding Blue Zones and the methodology behind them. In 2024, Newman published a preprint questioning potential errors in the establishment of such regions, suggesting many reported centenarians may not be authentic [70]. His analysis found that after birth certificates became standard in 1900 in the USA, the number of verified supercentenarians declined, indicating possible age misreporting. In Greece, 70% of alleged centenarians were deceased. Newman also identified irregularities in birth records and noted that Blue Zones are often in remote, low-income areas with unreliable data. This phenomenon is not unprecedented [44, 71]; similar inconsistencies were identified in Vilcabamba, Ecuador, a region also considered to be a Blue Zone during the 20th century. It was determined that the population's extreme longevity was due to exaggerated reported ages. Similar cases have been documented in Abkhazia (Caucasus) and the Hunza Valley in Pakistan, where the absence of reliable birth records also led to apparent cases of extreme longevity that were later invalidated [44, 71, 72].

### Factors related to longevity

‘Well-characterized’ Blue Zones (Ogliastra, Okinawa and Nicoya) by the scientific literature share characteristics that could explain the longevity of their inhabitants. Some of these features are also present in regions under investigation. Geographical isolation in these regions has led to higher levels of inbreeding, the impact of which on longevity is not yet fully understood. The Blue Zones' climate and geography, with moderate temperatures, constant sunlight, and varied topography, promote regular physical activity. Regular physical activity has been linked to better cardiovascular and cognitive health, as well as a lower risk of chronic diseases and longer survival [73-76].

Regarding diet, the focus on plant-based, low-meat, moderate-calorie foods is associated with longevity and reduced risk of all-cause mortality [77]. The Mediterranean diet in Ikaria and Ogliastra or the Japanese diet in Okinawa have been associated with increased longevity, although there is still insufficient evidence in this regard [27, 78]. It should be mentioned that it is not clear whether this dietary pattern responds to tradition, economic constraints or a combination of both factors. In addition, these regions lack advanced health services, indicating that extreme longevity seems to be more closely associated with lifestyle and self-sufficiency than with medical care.

Some studies have also identified genetic factors related to longevity. In the case of Okinawa, researchers found an association between a specific gene (FOXO3) and longevity, which has been confirmed by independent studies [43, 46]. Studies conducted by Willcox and Bendjilali concluded that, genetically speaking, the inhabitants of Okinawa constitute a distinct group from the rest of Japan's population [40, 48]. In the case of Martinique and Guadeloupe, the hypothesis explaining the longevity of their inhabitants is also genetic. The main hypothesis suggests a historical genetic selection, the extreme mortality during slavery may have favored ancestors with biological resilience, leading to the transmission of genes associated with longevity [67]. N1 (The Netherlands) residents may have also a greater genetic predisposition to longevity, evidenced by a favorable polygenic risk score and the presence of the apolipoprotein E-epsilon2 allele [33].

It is worth noting that some of the previously mentioned factors related to longevity are often based on small-scale or nonreplicated studies, such as the presence of genetic polymorphisms or the role of social networks. Therefore, some of the factors mentioned remain hypothesis-generating due to insufficient evidence.

The distribution of centenarians by sex varies across Blue Zones, but generally the number of women tends to exceed the number of men. However, in Ogliastra (Sardinia), an unusual balance between men and women has been observed [2, 6], unlike the typical higher female survival at advanced ages. While this pattern contrasts sharply with regions like Martinique and Guadeloupe (where all centenarians were women [67]), its consistency in Ogliastra highlights a possible regional specificity. Further studies are needed to explore whether demographic, cultural, or environmental factors drive this difference.

### Under-studied aspects of Blue Zones

Despite the growing literature on Blue Zones and the fact that some of them were defined more than 20 years ago, there is scarce scientific evidence on certain aspects.

The impact of socioeconomic status on longevity is largely unexplored. While the social gradient theory links higher socioeconomic status to longer life expectancy, Blue Zones have relatively low-income levels. Studies, including that by Cockerman et al. [79], suggest that Okinawa is contrary to the social gradient theory, and this may be applicable to other Blue Zones as well. Centenarians often maintain low incomes, even in retirement, which may influence their diet and encourage physical activity through food cultivation. This suggests that factors other than income may be more significant in determining longevity.

There is limited evidence on medication use and healthcare service frequency in Blue Zones. A study in Ikaria found low polypharmacy rates [26], but no other regions have explored this aspect. The occupations of centenarians remain understudied, though they are believed to have engaged in physically demanding outdoor work. While long-lived populations tend to have lower tobacco use and obesity rates, these factors have not been thoroughly examined.

It is worth mentioning that in recent years, the concept of non-naturally occurring Blue Zones has emerged. These projects aim to replicate conditions associated with longevity by intervening in social and lifestyle aspects. For instance, a project in Malvalaan, The Netherlands [80] and one in the Beach Cities in California, United States (www.bluezones.com/blue-zones-project-results-beach-cities-ca/), seek to promote physical activity, a balanced diet, and community cohesion through planned interventions. These projects often combine urban and special improvements.

### Advantages and limitations

The main advantage of this review is that it is the first study to comprehensively address the identification of Blue Zones, and the factors associated with the longevity of their population. In addition, it provides a critical assessment of whether there is sufficient evidence to officially consider these areas as Blue Zones. The main limitation is that several studies were identified from outside the literature search, despite extensive searches of several databases with broad inclusion criteria. This could be due to the indexing of articles, or the nomenclature used for Blue Zones. It has not been possible to combine the metrics related to longevity because not all identified Blue Zones have these metrics calculated or do not follow the same methodology, therefore they are not comparable. Future studies should calculate longevity metrics in the Blue Zones using a common method. Additionally, the concentration of identified articles from Blue Zones in Europe highlights potential geographical biases in longevity research. This imbalance may be due to unequal research infrastructure, funding disparities or differences in consistency on the date where registries were created. Regions such as Africa, South Asia or Latin America may also have extreme longevity regions and factors that promote longevity, but remain under-documented.

### Conclusions

The results of this review indicate that three regions — Okinawa, Ogliastra and Nicoya— have demographic and epidemiological studies that characterize them as Blue Zones. However, a loss of longevity advantage has been observed in some of these zones in recent decades, highlighting the need for continued research. There are also areas under investigation, such as Ikaria, Martinique/Guadeloupe, Cilento, a municipality in the Netherlands and Rugao, while others, such as Loma Linda or Menorca, do not meet sufficient scientific criteria or lack evidence to be designated as such. The absence of comprehensive studies on factors such as socioeconomic status, occupations, and utilization of medical services highlights the necessity for further research to better understand the determinants of longevity.

## Supplementary Materials

The Supplementary data can be found online at: www.aginganddisease.org/EN/10.14336/AD.2025.0461.
