# Supplementary material for: Blue Zones, an Analysis of Existing Evidence through a Scoping Review
Source: Aging Dis. 2025 May 18;17(3):1335–46. doi: 10.14336/AD.2025.0461 (PMC13061570; doi:10.14336/AD.2025.0461)
Supplement: Supplementary file 1 — The Supplementary data can be found online at: www.aginganddisease.org/EN/10.14336/AD.2025.0461. [file AD-17-3-1335-s.pdf]

SUPPLEMENTARY DATA

Blue Zones, an Analysis of Existing Evidence through a Scoping Review

Cristina Candal-Pedreira, Julia Rey-Brandariz, Lucía Martín-Gisbert, Ana Teijeiro, Guadalupe García, Alberto Ruano-Ravina, Mónica Pérez-Ríos

© 2025. Candal-Pedreira C et al. Published online at http://www.aginganddisease.org/EN/10.14336/AD.2025.0461

SUPPLEMENTARY DATA

SUPPLEMENTARY MATERIAL

Supplementary Table 1. Search strategy used in the scoping review.

Database	Search strategy
EMBASE<br>MEDLINE<br>PSYCINFO	((blue adj zone) or (blue adj zones) or LBZ).ti,ab. and (old or older or oldest or elderly or longevity or age or aged or ageing or aging or centennial or centennials or centenarian or centenarians).ti,ab,kw.
WOS	(TS=(((blue NEAR zone) or (blue NEAR zones) or LBZ) and (old or older or oldest or elderly or longevity or age or aged or ageing or aging or centennial or centennials or centenarian or centenarians))) OR TI=(((blue NEAR zone) or (blue NEAR zones) or LBZ) and (old or older or oldest or elderly or longevity or age or aged or ageing or aging or centennial or centennials or centanarian or centenarians))
SCOPUS	(TITLE-ABS-KEY ((blue W/0 zone) OR (blue W/0 zones) OR "LBZ") AND TITLE-ABS-KEY (old OR older OR oldest OR elderly OR longevity OR age OR aged OR ageing OR aging OR centennial OR centennials OR centenarian OR centen arians))

DETAILED DESCRIPTION OF THE INCLUDED REGIONS

Sardinia (Italy)

Identification

The first documentation of Sardinia as a region of notable longevity was in 1999, as reported in a study conducted by Akea (1). The study revealed that Sardinia exhibited a higher prevalence of centenarians compared to the rest of Europe (16.6 per 100,000 versus 10.0 per 100,000 in Europe). A notable finding was the disparity in the female-to-male ratio among the centenarian population, which was 2:1. This stands in contrast to the typical female-to-male ratio observed in this age group, which is 5:1. These two facts were most evident in 40 municipalities, especially in Nuoro, remote, mountainous area in Sardinia. Subsequent analysis of the study corroborated the age data of centenarians identified in prior studies and accurately identified an area where the observed number of centenarians was three times higher than expected (2).

Factors related to longevity

Ogliastra is located in the center-east of the island of Sardinia. The region is characterized by its mountainous topography, remote location, and prior to the mid-1990s challenging accessibility. These characteristics contributed to a population with low migration and inbreeding rates, leading to a decline in genetic variability. Additionally, it has been postulated that the differential exposure to infectious diseases may have influenced the population's immune response (3). One study found no association between genetic polymorphisms commonly associated with longevity in the Sardinian Blue Zone population (4).

The nutritional transition in Sardinia is widely reported to have occurred in 1950. A frugal diet based on self-cultivation of cereals, legumes, potatoes or the collection of wild fruits and honey, with a

© 2025. Candal-Pedreira C et al. Published online at http://www.aginganddisease.org/EN/10.14336/AD.2025.0461

low consumption of meat and dairy products, mainly derived from sheep's and goat's milk, highlighting the consumption of ricotta cheese was abandoned (5). With the nutritional transition, the diet changed, increasing the consumption of carbohydrates, olive oil, meat and fresh fruit and decreasing the consumption of bacon, legumes and vegetables (6). One review points to the impact of "famine foods" that were relied on due to food shortages at certain periods (7). One study showed association between mild overweight and improved survival in the nonagenarians of the Blue Zone (8). In this region, it is worth mentioning the preservation of traditional cultural practices and familial bonds that has been linked to enhanced mental well-being (9) and the adoption of active lifestyles into advanced age that has been associated with preserved cognitive capacity (10-12) or improvement of mental health (13-15).

Calculated indicators

In 2012, a study was published with the objective of verifying the persistence of high longevity in areas of Sardinia. The Extreme Longevity Index (ELI) was calculated. The analysis of data from 1992, 2001, and 2012 revealed that no specific region of Sardinia emerged as significantly distinguished from others. The highest observed longevity values were identified in Barbagia de Ollolai (16).
A study that assessed the differences in mortality according to birth cohort and sex in Villagrande Strisaili, a village identified within the Blue Zone, concluded that there is no trend in the survival of the cohorts under study. The study found that the differences are greater among the male cohorts, and that the 1902-1911 cohort has the highest survival at advanced ages (17).

Okinawa (Japan)

Identification

The first article reporting the exceptional longevity of the Okinawan population was published in 1976 by Kagawa et al. (18), following the publication of the 1975 census, which enumerated the 37 centenarians living in Okinawa (prevalence of 35.5 per million compared to 5.1 per million in the rest of Japan). Shortly thereafter, the Japanese Ministry of Health published a list ranking Okinawa as the prefecture with the best longevity indicators: life expectancy at birth, life expectancy at age 65, and the proportion of nonagenarians and centenarians in the total population for men and women (19). An article published by Willcox et al. in 2008 validated the age of 8% of the island's centenarians, confirming the high prevalence of centenarians (20).

Factors related to longevity

After Okinawa was identified as the place with the most centenarians in the world, several studies were carried out with the aim of discovering factors that could influence this fact. In 1975, the "Okinawa Centenarian Study", funded by the Japanese Ministry of Health, was initiated and is still active. The aim was to study dietary, lifestyle, genetic, psychological and spiritual, social and behavioral factors that may be associated with longevity. Since its inception, the study has included more than 3,000 Okinawan centenarians. The main result of this study was the association of a specific gene (FOXO3) with longevity, which has been confirmed by other independent studies (21, 22). Studies by Willcox et al. and Bendjilali et al. concluded that the inhabitants of Okinawa were genetically distinct from the rest of the Japanese population (23, 24).

In addition to studying the effect of genetics and family history, nutrition is one of the most studied factors regarding longevity in Okinawans (24, 25). In fact, the low mortality rates in Okinawa are believed to be due to the rice and vegetable-based diet followed by its inhabitants (26) (27-29). The

© 2025. Candal-Pedreira C et al. Published online at http://www.aginganddisease.org/EN/10.14336/AD.2025.0461

traditional Okinawan diet is low in calories, but nutritionally dense in vitamins, minerals and phytonutrients (30). A diet consisting of pork (low in saturated fats) and vegetables (high levels of antioxidants) has been identified as a protective factor against various diseases, including cardiovascular disease and certain types of cancer (31). Daily physical activity, mainly due to agriculture, also seems to play a role in the longevity of Okinawans (32). In addition, cars arrived later than in other places.

Other factors, although less studied, are the warm climate, cultural, social and religious factors (33).

Indicators

The absolute number of centenarians in Okinawa increased from 37 in 1975 to 1271 in 2022 (33). This increase was predominantly observed among the female population; in the 1980s, there were 400 female centenarians for every 100 male centenarians, while in 2006, this number increased to 800. The male-to-female ratio continued to decrease until 2009 and then increased slightly and remained stable. Okinawa is therefore characterized primarily by the exceptional longevity of women.

Okinawa maintained the top position in life expectancy among Japan's 47 prefectures until the end of the 20th century. Around 2020, Okinawa's life expectancy matched Japan's for women. In men, this happened in 2000 and currently Japan's life expectancy exceeds Okinawa's.

Since 1975, Okinawa's ELI has always remained above that of Japan and follows an upward trend (albeit with fluctuations). However, in recent years, mortality rates in Okinawa have increased faster than those in Japan.

All these data conclude that Okinawa's advantage in terms of longevity and life expectancy is no longer as evident as it was in the last century. Generations born in Okinawa before World War II were better off in terms of mortality and longevity than their peers born elsewhere in Japan. This situation gradually disappeared until those born in 1940. From that year onwards, the opposite was observed, i.e., those born elsewhere in Japan presented a more favorable situation in terms of mortality. The causes of this change may be the occidentalizing of Okinawa during the U.S. occupation during and after World War II, which modified living habits and diet. This was mirrored by an increase in adult cancer, heart disease and cerebrovascular disease. An increase in stress in younger generations is also reported (34).

Nicoya (Costa Rica)

Identification

Nicoya is composed of multiple cantons, of which five are situated within the province of Guanacaste, forming the renowned Blue Zone (Nicoya, Santa Cruz, Carrillo, Nandayure, and Hojancha). According to the Electoral Roll of the Supreme Electoral Tribunal, the region had 58 centenarians in 2023. In 2017, the population of centenarians was distributed with a male-to-female ratio of 1:1.38 (3).

The Nicoya peninsula was first mentioned as a place of high longevity in 2005 during a conference on the CRELES (Costa Rican Longevity and Healthy Aging Study) project. Since then, there have been a few studies analyzing several variables in the population of the Nicoya peninsula (35-38).

Factors related to longevity

Studies on this population found that the population of the Nicoya peninsula had fewer physical and mental disabilities than the rest of the residents of Costa Rica (37) and better indicators of cardiovascular and metabolic risk (37). Thus, residents of the Nicoya peninsula have lower cholesterol

© 2025. Candal-Pedreira C et al. Published online at http://www.aginganddisease.org/EN/10.14336/AD.2025.0461

levels, lower waist circumference, lower body mass index and lower blood pressure than the population residing in the rest of Costa Rica (37). A relatively small percentage of the population in Nicoya is affected by overweight and obesity, and the local population exhibits a higher level of physical activity compared to other populations in Costa Rica (39). In addition, it is a population with a lower prevalence of chronic diseases such as diabetes, COPD, depression, ischemic heart disease (36) and a lower consumption of medications for chronic conditions (36, 37).

An additional factor that was the subject of analysis in this population was the diet, which includes foods such as rice, beans, meat, fish, and chicken (37, 39, 40). It has been observed that neither the consumption of red meat nor the consumption of processed and fast food is a common dietary practice among this population (37, 39). In addition, they also drink less milk daily and so have a lower intake of calcium (37). The diet of the Nicoya peninsula population is light but higher in calories, with a higher consumption of carbohydrates, trans and saturated fats, protein, and dietary fiber (37). Some authors indicate that Nicoyans are more likely to maintain food customs and traditions because they are more isolated from large urban centers where nutritional transitions are occurring due to globalization (39).

A range of additional factors have been identified, including those associated with genetics or the environment. Research examining the epigenetics of Nicoya peninsula residents revealed that this population exhibited reduced levels of stress and found out they had longer telomeres (37, 38) and higher levels of dehydroepiandrosterone sulfate (DHEAS) (38). On the other hand, other studies have also observed that the immunological profile of individuals from the Nicoya peninsula was younger than that of the rest of Costa Rica (41). In terms of environmental factors, the Nicoya peninsula exhibits low noise levels, favorable air quality, high levels of luminosity, and high temperatures that promote social interaction among residents (40).

<u>Indicators</u>

Regarding mortality rates, Rosero-Bixby et al. (37) observed a lower relative mortality rate in men aged 60 and over during the 1990-2011 period in the region, compared to the national average. On the other hand, in men, there was also a lower mortality due to cardiovascular diseases, which was not observed in women or when the cause of death analyzed was cancer (37). The probability of becoming a centenarian was estimated to be higher in men than in women (37, 39).

Studies of cohorts born before 1930 support high longevity in Nicoya, although this characteristic is decreasing due to a cohort effect. People born before 1930 showed exceptional longevity, but this phenomenon is no longer observed in more recent cohorts. In addition, the area of extreme longevity in Nicoya has decreased from a population of about 207,000 people in five cantons to a smaller area of about 25,000 inhabitants, mainly in the canton of Hojancha and areas south of the city of Nicoya (35).

Ikaria (Greece)

<u>Identification</u>

A higher life expectancy was observed in Ikaria compared to the average life expectancy of the rest of Greece. Consequently, since 2009, the population between 65 and 100 years of age has been the subject of study through the IKARIA study (42).

<u>Factors related to longevity</u>

Several studies have been conducted to analyze the sociodemographic characteristics of the population of Ikaria (42-46). The results show that the population has a low socioeconomic (43, 45) and

© 2025. Candal-Pedreira C et al. Published online at http://www.aginganddisease.org/EN/10.14336/AD.2025.0461

educational level, since 20.3% had not completed elementary school, and the illiteracy rate was 10.1% (43).

In this population, social activities emerged as the primary factor under analysis. The Ikarian population displays a notable degree of family solidarity, characterized by the tendency of older individuals to reside with family members rather than in institutionalized settings (43-45).

Furthermore, the level of social interaction is remarkably elevated, as evidenced by the maintenance of daily social contacts and active participation in social and religious events, such as the Panigiria (44).

The factors analyzed also include diet, physical activity and sleep quality. It has been observed that the population of Ikaria has a high adherence to the Mediterranean diet (44) as they have a high consumption of oil, fruits, vegetables and potatoes per week and a lower consumption of sweets, red meat, cereals and fish (43). In addition, physical activity is common in this population, especially in men (43, 44). It was observed that they had good quality sleep, especially men (45) and that the majority (70%) took a nap during the day (44).

The presence of diseases and risk factors was another aspect analyzed in some studies. The population of Ikaria had cardiovascular risk factors such as hypertension, diabetes, overweight or obesity, smoking and alcohol consumption (43, 45). However, they had a lower prevalence of multimorbidity (46), polypharmacy rates (45), or depression (43, 45).

<u>Indicators</u>

There are few studies in the Ikarian population that analyze mortality indicators (47) and no studies have been found that analyze ELI. However, these studies mention that census data and age-at-death statistics were collected from the Hellenic Statistical Authority and compared with individual information extracted from a local administrative register containing demographic data of Greek citizens at the municipal level. For the oldest, as no birth records were found, the extinct cohort method was used to estimate longevity, with ages being validated by interviews with people aged 90 years and older in the northwest of the island (48).

Cilento (Italy)

<u>Identification</u>

There are several studies carried out on Cilento, trying to classify it as a Blue Zone. One study compares the characteristics of Cilento with the other Blue Zones, to analyze differences in diet and lifestyle (2024) (49) and the other study tries to delimit Cilento as a Blue Zone from an epidemiological and geographical point of view (2022) (50). A third article attempts to define whether there is any relationship between the characteristics of drinking water (pH, hardness, mineral content) and greater longevity at the municipal level. The article concludes that drinking water is mostly alkaline in nature and that there is an absence of heavy metals. The authors suggest that a potential correlation exists between medium-to-high hardness levels and enhanced longevity (51).

<u>Factors related to longevity and indicators</u>

In one of the studies, six indicators of longevity are developed, all of them well defined through previous bibliographic sources (50). The indicators are as follows: 1) centenarian rate (number of centenarians per 10,000 inhabitants); 2) aging trend (population over 65 years of age among total population); 3) ratio of population over 85 years of age (population over 85 years of age among total population); 4) ratio of population over 90 years of age (population over 90 years of age among total

© 2025. Candal-Pedreira C et al. Published online at http://www.aginganddisease.org/EN/10.14336/AD.2025.0461

population); 5) longevity index (ratio of population over 90 years of age among population over 65 years of age); 6) centenarian index (ratio of centenarians among population over 90 years of age).
In addition to the aforementioned factors, the researchers also considered other environmental factors (altitude, average temperature and humidity), its status as a World Heritage Site, and as a hinterland. The authors performed a relatively complex analysis, describing the zones by spatial autocorrelation. The researchers found a significant correlation between all the indicators, concluding that some of the factors indicated may contribute to the greater longevity observed.

Martinique/Guadeloupe (overseas department of France)

Identification
A study published in 2020 identifies Guadeloupe and Martinique as potential Blue Zones, as evidenced by their high concentration of supercentenarians, defined as individuals over 110 years of age (52). The study validates the ages of supercentenarians and concludes that they are not due to recording errors.
Factors associated with longevity
Factors associated with longevity on these islands include possible genetic selection derived from slavery. During that period, only the strongest and most resistant individuals survived and had descendants, which may have favored genes related to longevity. In addition, it was observed that the mothers of supercentenarians had high fertility (average of 8 children), while supercentenarians themselves tended to have fewer descendants (average of 3 children). Their siblings also showed a longer life expectancy compared to the general population, reinforcing the hypothesis of a genetic component.
Indicators
Between 1988 and 2016, the prevalence of supercentenarian deaths observed in Guadeloupe and Martinique is 7-8 times higher than in metropolitan France. Guadeloupe and Martinique, show a high supercentenarian death prevalence (21 and 24 per million inhabitants, respectively), exceeding rates in metropolitan France (3 per million).

Menorca (Spain)

Identification
The island of Menorca has only one published study with the objective of analyzing whether the island meets the characteristics to be defined as a Blue Zone (53). The study delimits a target area, obtains birth records for a 20-year period, and quantifies births to identify still-living individuals. An exhaustive identification of deceased centenarians is performed, and the age of living centenarians is validated, whether or not they reside in the target area. Subsequently, the ELI is calculated.
Indicators
The study concludes that, to obtain a stable index, at least 100,000 inhabitants would be needed. The male-to-female ratio is also calculated for people over 100 years of age and a higher proportion of males is observed, although numerical instability prevents definitive conclusions from being drawn. Finally, the authors conclude that there does not seem to be a Blue Zone in Es Migjorn Gran or in the surrounding municipalities.

Rugao (China)

© 2025. Candal-Pedreira C et al. Published online at http://www.aginganddisease.org/EN/10.14336/AD.2025.0461

SUPPLEMENTARY DATA

Identification

A region located in northeast China called Rugao is being studied to establish a possible Blue Zone. This region is identified as the longest-lived region in the country. For this reason, the Rugao Longevity and Ageing Study (RuLAS) began between 2007 and 2008 (54), initiated by Fudan University. This is a cohort study in which in one arm are subjects aged 95 years and older and the other arm includes participants aged 70-84 years. This study has given rise to several publications in recent years, although they do not focus on identifying factors related to longevity (55-57).
Three other studies have been identified in this area, all of them focused on geological and ecological aspects of the area, without classifying this area as a Blue Zone (58-60).

Indicators

Huang et al. conducted a study between 2011 and 2015 with the objective of assessing the longevity in the area under study. To do so, they used different statistical indices, including the ELI. The researchers concluded that there is a stable longevity zone in the center of the region under study and that in this area people have an easier time living to 90-99 years, but there are fewer cases of people reaching 100 years. Furthermore, the researchers observed a progressive decline in the male-to-female ratio with increasing age (61).

Loma Linda (California, USA)

Identification

Dan Buettner identified Loma Linda as a Blue Zone in a 2005 National Geographic cover story. It is now listed as a Blue Zone on the corporate website for the Blue Zone LLC brand created by Dan Buettner and in entertainment media, most notably a Netflix documentary miniseries.

The population of Loma Linda is strongly influenced by the Seventh-day Adventist community located there. The community's members eat a predominantly vegetarian diet, do not drink alcohol, engage in moderate physical activity on a regular basis, and pay special attention to spirituality (62).

There are no studies in the scientific literature on longevity in Loma Linda, nor is there any publication showing evidence of the indicators used to identify Loma Linda as a Blue Zone.

A municipality in the Netherlands

Identification

Different municipalities in the Netherlands are being studied under an ongoing longitudinal study "Longitudinal Aging Study Amsterdam". It covers waves since 1992/1993 and the results, performed in 2019, were published in 2024 (63). Of this, one municipality (unidentified in the article) fulfilled all three criteria to be considered as a Blue Zone (cumulative number of exceptionally longevous participants, the highest life expectancy and the most stable population). This municipality has a cumulative proportion of exceptional long-living participants of 8.3%. The characteristics found were that individuals of this zone had an education 1.5-year shorter than other municipalities and rest of provinces of the country. Unlike other Blue Zones, when comparing this municipality among the rest of the provinces of the Netherlands, body mass index and waist circumference were slightly larger, and they consumed vegetables less often. Also, they walked less often but they biked for longer. In this region, more emotional support was received.

© 2025. Candal-Pedreira C et al. Published online at http://www.aginganddisease.org/EN/10.14336/AD.2025.0461

SUPPLEMENTARY DATA

References

1. Deiana L, Ferrucci L, Pes GM, Carru C, Delitala G, Ganau A, et al. AKEntAnnos. The Sardinia Study of Extreme Longevity. Aging (Milano). 1999;11(3):142-9.
2. Poulain M, Pes GM, Grasland C, Carru C, Ferrucci L, Baggio G, et al. Identification of a geographic area characterized by extreme longevity in the Sardinia island: The AKEA study. Experimental Gerontology. 2004;39(9):1423-9.
3. Soloski MJ, Poulain M, Pes GM. Does the trained immune system play an important role in the extreme longevity that is seen in the Sardinian blue zone? Frontiers in aging. 2022;3:1069415.
4. Errigo A, Dore MP, Mocci G, Pes GM. Lack of association between common polymorphisms associated with successful aging and longevity in the population of Sardinian Blue Zone. Sci. 2024;14(1):30773.
5. Pes GM, Tolu F, Errigo A, Concu D, Chambre D, Poulain M. Genetic and non-genetic factors associated with population longevity in Sardinia. European Geriatric Medicine. 2014;5(SUPPL. 1):S148.
6. Pes GM, Poulain M, Errigo A, Dore MP. Evolution of the dietary patterns across nutrition transition in the sardinian longevity blue zone and association with health indicators in the oldest old. Nutrients. 2021;13(5):1495.
7. Wang C, Murgia MA, Baptista J, Marcone MF. Sardinian dietary analysis for longevity: a review of the literature. J Ethnic Food. 2022;9(1).
8. Pes GM, Errigo A, Dore MP. Association between Mild Overweight and Survival: A Study of an Exceptionally Long-Lived Population in the Sardinian Blue Zone. J. 2024;13(17):09.
9. Hitchcott PK, Fastame MC, Ferrai J, Penna MP. Psychological Well-Being in Italian Families: An Exploratory Approach to the Study of Mental Health Across the Adult Life Span in the Blue Zone. Europe's journal of psychology. 2017;13(3):441-54.
10. Carta E, Riccardi A, Marinetto S, Mattivi S, Selini E, Pucci V, et al. Over ninety years old: Does high cognitive reserve still help brain efficiency? Psychological research. 2024;88(2):678-83.
11. Fastame MC, Mulas I, Ruiu M. Associations between migration experience and perceived mental health in optimal ageing: Evidence from the Sardinian Blue Zone. International journal of psychology : Journal international de psychologie. 2022;57(2):271-8.
12. Carboni D, Mazza G. Sardinia between transhumance and the Blue Zone. Socio-spatial dynamics, rural lifestyle and diet of a geographical area of exceptional longevity: Ogliastra. Documenti Geografici. 2023(3):101-18.
13. Fastame MC, Mulas I, Pau M. Mental health and motor efficiency of older adults living in the Sardinia's Blue Zone: A follow-up study. International Psychogeriatrics. 2021;33(12):1277-88.
14. Ruiu M, Carta V, Deiana C, Fastame MC. Is the Sardinian Blue Zone the New Shangri-La for mental health? Evidence on depressive symptoms and its correlates in late adult life span. Aging Clinical and Experimental Research. 2022;34(6):1315-22.
15. Brandas B, Fastame MC. Psychological markers of successful ageing in Blue Zones: Experimental evidence and future perspectives. Giornale Italiano di Psicologia. 2023;50(4):699-728.
16. Orrù A, Brizzi M, Sanna E. There is a geographical area of extreme longevity (Blue Zone) in Sardinia? J Biol Res. 2012;85(1):139-40.
17. Salaris L. Differential mortality in a long-living community in Sardinia (Italy): a cohort analysis. Journal of biosocial science. 2015;47(4):521-35.
18. Kagawa Y, Ishiguro M, Okuno M, Tsukiji H, Terakado M, Iwamuro S, et al. Area of longevity in Japan in 1976. Jpn J Nutr. 1976; 34: 163–172
19. Miyagi S, Iwama N, Kawabata T, Hasegawa K. Longevity and diet in Okinawa, Japan: the past, present and future. Asia Pac J Public Health. 2003;15 Suppl:S3-9.
20. Willcox DC, Willcox BJ, He Q, Wang NC, Suzuki M. They really are that old: a validation study of centenarian prevalence in Okinawa. J Gerontol A Biol Sci Med Sci. 2008;63(4):338-49.

21. Morris BJ, Willcox DC, Donlon TA, Willcox BJ. FOXO3: A Major Gene for Human Longevity--A Mini-Review. Gerontology. 2015;61(6):515-25.
22. Willcox BJ, Donlon TA, He Q, Chen R, Grove JS, Yano K, et al. FOXO3A genotype is strongly associated with human longevity. Proc Natl Acad Sci U S A. 2008;105(37):13987-92.
23. Willcox BJ, Willcox DC, He Q, Curb JD, Suzuki M. Siblings of Okinawan centenarians share lifelong mortality advantages. J Gerontol A Biol Sci Med Sci. 2006;61(4):345-54.
24. Bendjilali N, Hsueh WC, He Q, Willcox DC, Nievergelt CM, Donlon TA, et al. Who are the Okinawans? Ancestry, genome diversity, and implications for the genetic study of human longevity from a geographically isolated population. J Gerontol A Biol Sci Med Sci. 2014;69(12):1474-84.
25. Suzuki M, Mori H, Asoto T, Sakugawa H, Ishii T, Hosoda Y. Medical research upon centenarians in Okinawa—case controlled study of family history as hereditary influence on longevity. Jpn J Geriatr. 1985; 22: 457–467. .
26. Willcox BJ, Willcox DC. Caloric restriction, caloric restriction mimetics, and healthy aging in Okinawa: controversies and clinical implications. Curr Opin Clin Nutr Metab Care. 2014;17(1):51-8.
27. Akisaka M, Asato L, Chan YC, Suzuki M, Uezato T, Yamamoto S. Energy and nutrient intakes of Okinawan centenarians. J Nutr Sci Vitaminol (Tokyo). 1996;42(3):241-8.
28. Chan YC, Suzuki M, Yamamoto S. Dietary, anthropometric, hematological and biochemical assessment of the nutritional status of centenarians and elderly people in Okinawa, Japan. J Am Coll Nutr. 1997;16(3):229-35.
29. Sho H. History and characteristics of Okinawan longevity food. Asia Pac J Clin Nutr. 2001;10(2):159-64.
30. Willcox DC, Scapagnini G, Willcox BJ. Healthy aging diets other than the Mediterranean: a focus on the Okinawan diet. Mech Ageing Dev. 2014;136-137:148-62.
31. Willcox DC, Willcox BJ, Todoriki H, Suzuki M. The Okinawan diet: health implications of a low-calorie, nutrient-dense, antioxidant-rich dietary pattern low in glycemic load. J Am Coll Nutr. 2009;28 Suppl:500S-16S.
32. Akisaka M, Suzuki M. The bone density and activities of daily living in Okinawa centenarians. Hong Kong J Gerontol. 1996; 10: 453–457. .
33. Poulain M, Herm A. Exceptional longevity in Okinawa: Demographic trends since 1975. J Intern Med. 2024;295(4):387-99.
34. Ryall J. What's behind Okinawa's falling life expectancy? DW News. 2022. Accessed on 15 Oct 2023 at https://p.dw.com/p/4CVyC
35. Rosero-Bixby L. The vanishing advantage of longevity in Nicoya, Costa Rica: A cohort shift. Demogr Res. 2023;49:723-36.
36. Madrigal-Leer F, Martinez-Montandon A, Solis-Umana M, Helo-Guzman F, Alfaro-Salas K, Barrientos-Calvo I, et al. Clinical, functional, mental and social profile of the Nicoya Peninsula centenarians, Costa Rica, 2017. Aging Clinical and Experimental Research. 2020;32(2):313-21.
37. Rosero-Bixby L, Dow WH, Rehkopf DH. The Nicoya region of Costa Rica: a high longevity island for elderly males. Vienna Yearb Popul Res. 2013;11:109-36.
38. Rehkopf DH, Dow WH, Rosero-Bixby L, Lin J, Epel ES, Blackburn EH. Longer leukocyte telomere length in Costa Rica's Nicoya Peninsula: a population-based study. Exp Gerontol. 2013;48(11):1266-73.
39. Momi-Chacon A, Capitan-Jimenez C, Campos H. Dietary habits and lifestyle among long-lived residents from the Nicoya Peninsula of Costa Rica. Rev Hisp Cienc Salud. 2017; 3(2): 53-60.
40. Hennia María Cavallini Solano, Gilbert Brenes Camacho, Juan Carlos Vargas Aguilar, María Fernanda Abarca Jiménez. El entorno socioambiental de las redes sociales de apoyo de personas longevas en la Península de Nicoya, Costa Rica, desde la perspectiva del modelo básico de capacidades. Anales en Gerontología 2022, ISSN 1659-0813, Vol. 14, Nº. 14, págs. 96-137.
41. McEwen BS. Neurobiological and Systemic Effects of Chronic Stress. Chronic Stress (Thousand Oaks). 2017;1.
42. Stefanadis CI. Unveiling the secrets of longevity: the Ikaria study. Hellenic J Cardiol. 2011;52(5):479-80.
43. Panagiotakos DB, Chrysohoou C, Siasos G, Zisimos K, Skoumas J, Pitsavos C, et al. Living longer, living better; Which are the secrets? the Ikaria study. European Heart Journal. 2011;32(SUPPL. 1):721.

44. Legrand R, Nuemi G, Poulain M, Manckoundia P. Description of lifestyle, including social life, diet and physical activity, of people =90 years living in Ikaria, a longevity blue zone. International Journal of Environmental Research and Public Health. 2021;18(12):6602.
45. Legrand R, Manckoundia P, Nuemi G, Poulain M. Assessment of the Health Status of the Oldest Olds Living on the Greek Island of Ikaria: A Population Based-Study in a Blue Zone. Current gerontology and geriatrics research. 2019;2019:8194310.
46. Foscolou A, Chrysohoou C, Dimitriadis K, Masoura K, Vogiatzi G, Gkotzamanis V, et al. The Association of Healthy Aging with Multimorbidity: IKARIA Study. Nutrients. 2021;13(4).
47. Chrysohoou C, Pitsavos C, Lazaros G, Skoumas J, Tousoulis D, Stefanadis C, et al. Determinants of All-Cause Mortality and Incidence of Cardiovascular Disease (2009 to 2013) in Older Adults: The Ikaria Study of the Blue Zones. Angiology. 2016;67(6):541-8.
48. Poulain M, Herm A, Errigo A, Chrysohoou C, Legrand R, Passarino G, et al. Specific features of the oldest old from the Longevity Blue Zones in Ikaria and Sardinia. Mech Ageing Dev. 2021;198:111543.
49. Aliberti SM, Donato A, Funk RHW, Capunzo M. A Narrative Review Exploring the Similarities between Cilento and the Already Defined "Blue Zones" in Terms of Environment, Nutrition, and Lifestyle: Can Cilento Be Considered an Undefined "Blue Zone"? Nutrients. 2024;16(5).
50. Aliberti SM, De Caro F, Funk RHW, Schiavo L, Gonnella J, Boccia G, et al. Extreme Longevity: Analysis of the Direct or Indirect Influence of Environmental Factors on Old, Nonagenarians, and Centenarians in Cilento, Italy. Int J Environ Res Public Health. 2022;19(3).
51. Aliberti SM, Funk RHW, Ciaglia E, Gonnella J, Giudice A, Vecchione C, et al. Old, Nonagenarians, and Centenarians in Cilento, Italy and the Association of Lifespan with the Level of Some Physicochemical Elements in Tap Drinking Water. Nutrients. 2023;15(1).
52. Vallin J. Why are supercentenarians so frequently found in French Overseas Departments? The cases of Guadeloupe and Martinique. Genus. 2020;76(1):26.
53. Soriano JB, Fernandez Vazquez S, Carretero S, Puga Gonzalez MD, Soriano C, Romaguera D, et al. Description of extreme longevity in the Balearic Islands: Exploring a potential Blue Zone in Menorca, Spain. Geriatr Gerontol Int. 2014;14(3):620-7.
54. Liu Z, Wang Y, Zhang Y, Chu X, Wang Z, Qian D, et al. Cohort Profile: The Rugao Longevity and Ageing Study (RuLAS). Int J Epidemiol. 2016;45(4):1064-73.
55. Zhi T, Wang Q, Liu Z, Zhu Y, Wang Y, Shi R, et al. Body mass index, waist circumference and waist-hip ratio are associated with depressive symptoms in older Chinese women: results from the Rugao Longevity and Ageing Study (RuLAS). Aging Ment Health. 2017;21(5):518-23.
56. Liu ZY, Wang ZD, Li LZ, Chu XF, Zhu YS, Shi JM, et al. Association of CRP gene polymorphisms with CRP levels, frailty and co-morbidity in an elderly Chinese population: results from RuLAS. Age Ageing. 2016;45(3):360-5.
57. Liu ZY, Shen YY, Ji LJ, Jiang XY, Wang XF, Shi Y. Association between serum beta2-microglobulin levels and frailty in an elderly Chinese population: results from RuLAS. Clin Interv Aging. 2017;12:1725-9.
58. Zhao Y., Xu X., Darilek J., Huang B., Sun W., Shi X. Spatial variability assessment of soil nutrients in an intense agricultural area, a case study of Rugao County in Yangtze River Delta Region, China. Environ. Geol. 2009;57:1089–1102. doi: 10.1007/s00254-008-1399-5.
59. Sun W., Huang B., Zhao Y., Shi X., Darilek J., Deng X., Wang H., Zou Z. Spatial variability of soil selenium as affected by geologic and pedogenic processes and its effect on ecosystem and human health. Geochem. J. 2009;43:217–225. doi: 10.2343/geochemj.1.0019.
60. Huang B., Zhao Y., Sun W., Yang R., Gong Z., Zou Z., Feng D., Su J. Relationships between distributions of longevous population and trace elements in the agricultural ecosystem of Rugao County, Jiangsu, China. Environ. Geochem. Health. 2009;31:379–390. doi: 10.1007/s10653-008-9177-6.
61. Huang Y, Jacquez GM. Identification of a blue zone in a typical Chinese longevity region. International Journal of Environmental Research and Public Health. 2017;14(6):571.
62. Buettner D, Skemp S. Blue Zones: Lessons From the World's Longest Lived. Am J Lifestyle Med. 2016;10(5):318-21.

63. Deeg DJH, van Tilburg T, Visser M, Braam A, Stringa N, Timmermans EJ. Identification of a "Blue Zone" in the Netherlands: A Genetic, Personal, Sociocultural, and Environmental Profile. Gerontologist. 2024;64(11):01.

© 2025. Candal-Pedreira C et al. Published online at http://www.aginganddisease.org/EN/10.14336/AD.2025.0461
